# MiR-146a Regulates Migration and Invasion by Targeting NRP2 in Circulating-Tumor Cell Mimicking Suspension Cells

**DOI:** 10.3390/genes12010045

**Published:** 2020-12-30

**Authors:** Yeojin Do, Jin Gu Cho, Ji Young Park, Sumin Oh, Doyeon Park, Kyung Hyun Yoo, Myeong-Sok Lee, Byung Su Kwon, Jongmin Kim, Young Yang

**Affiliations:** 1 Division of Biological Sciences, Sookmyung Women’s University, Seoul 04310, Korea; yeojin0764@sookmyung.ac.kr (Y.D.); jgcho84@sookmyung.ac.kr (J.G.C.); pakgy1018@sookmyung.ac.kr (J.Y.P.); soh@sookmyung.ac.kr (S.O.); pdy1748@sookmyung.ac.kr (D.P.); khryu@sookmyung.ac.kr (K.H.Y.); mslee@sookmyung.ac.kr (M.-S.L.); 2 Research Institute for Women’s Health, Sookmyung Women’s University, Seoul 04310, Korea; 3 Department of Obstetrics and Gynecology, Kyung Hee University Medical Center, 23, Seoul 02447, Korea

**Keywords:** circulating-tumor cell, metastasis, MicroRNA, neuropilin2 (NRP2), semaphorin3C (SEMA3C), suspension cell

## Abstract

Cancer metastasis is the primary cause of cancer-related death and metastatic cancer has circulating-tumor cells (CTCs), which circulate in the bloodstream before invading other organs. Thus, understanding the precise role of CTCs may provide new insights into the metastasis process and reduce cancer mortality. However, the molecular characteristics of CTCs are not well understood due to a lack of number of CTCs. Therefore, suspension cells were generated from MDA-MB-468 cells to mimic CTCs, and we investigate the microRNA (miRNA)-dependent molecular networks and their role in suspension cells. Here, we present an integrated analysis of mRNA and miRNA sequencing data for suspension cell lines, through comparison with adherent cells. Among the differentially regulated miRNA–mRNAs axes, we focus on the miR-146a-Neuropilin2 (NRP2) axis, which is known to influence tumor aggressiveness. We show that miR-146a directly regulates NRP2 expression and inhibits Semaphorin3C (SEMA3C) signaling. Functional studies reveal that miR-146a represses SEMA3C-induced invasion and proliferation by targeting NRP2. Finally, high-NRP2 is shown to be associated with poor outcomes in breast cancer patients. This study identifies the key role of the miR-146a–NRP2 signaling axis that is critical for the regulation of migration and invasion in CTC-mimicking cells.

## 1. Introduction

Breast cancer is the most common cancer in women [1]. According to relative survival rates for female breast cancer by stage at diagnosis during 2008 through 2014, the survival rate of distant-cancer is 27% compared to 90% of all-stage breast cancer patients [2]. Because metastasis is the primary cause of death in cancer patients, research interest into metastasis suppression has been increasing. Circulating tumor cells (CTCs) form clusters in the bloodstream and act as metastatic precursors. Metastatic breast cancer patients who exhibit increased numbers of CTCs are given a poor prognosis. Therefore, CTC count is considered to be a prognostic biomarker for metastasis, recurrence, etc. [3,4]. However, because CTCs exist in low concentrations in peripheral blood cells, they are difficult to isolate and investigate [5]. To overcome these limitations, we developed CTC-mimicking MDA-MB-468 suspension cell lines [6]. MDA-MB-468-adherent cells were maintained and cultured in suspension, to imitate the molecular characteristics of CTCs. The suspension cells expressed EpCAM and cytokeratins, which are used as CTC markers when sorting CTCs in the bloodstream [7,8]. Moreover, the suspension cells exhibited an enhanced invasion capacity in orthotopic xenograft experiments and vimentin-staining experiments involving lung and liver tissue sections; this indicates that suspension cells have a higher metastatic potential than adherent cells. Therefore, suspension cells act as a CTC model, exhibiting enhanced metastatic abilities and epithelial CTC markers despite floating in a medium. In this study, to determine the molecular network and identify ways of inhibiting CTCs, we used CTC-mimicking suspension cells.

Non-coding small RNAs, referred to as microRNAs (miRNAs), play a key regulatory role in gene expression at the post-transcriptional level. A major action mechanism of miRNAs is in inhibiting the expression of target messenger RNAs (mRNAs) by directly binding to the 3′ untranslated region (3′UTR) of the targets [9,10]. In the tumor microenvironment, aberrant miRNA expression can be used to identify cancer characteristics. MiRNAs are regarded as oncogenes or tumor suppressors, which influence metastasis and cancer stemness [11]. Restoration of the miR-146a expression reduces its migration and invasion capacities in hepatocellular cancer (HCC) in vitro and in vivo. In addition, miR-146a is associated with metastatic HCC patients, suggesting that miR-146a expression levels are lower in HCC patients with metastasis than those without it [12]. In breast cancers, injecting miR-146a-expressing MDA-MB-231 cells inhibits pulmonary metastasis by 69% in athymic mice, and miR-146a represses the proliferation, migration, and invasion of MCF7 and MDA-MB-453 cells [13,14]. While the importance of investigating the miRNAs associated with various cancers is increasing, the role of miRNAs in CTCs is largely unknown.

Neuropilins (NRP1 and 2) are neuronal receptors that participate in axon guidance by interacting with the class-3 semaphorin (SEMA3) and vascular endothelial growth factor (VEGF) family [15]. NRP2 is also involved in oncological processes such as angiogenesis and metastasis. The knockdown of NRP2 impairs migration and invasion in hepatocellular carcinoma (e.g., 3sp and SNU-499) cell lines [16]. In H358 lung cancer cells, reducing NRP2 represses the formation of tumorspheres [17]. Increased NRP2 expression tends to be associated with lymph node metastasis and poor prognoses in breast cancer patients [18]. SEMA3C, one of the NRP2 ligands, contributes to tumorigenesis and metastasis. Silencing SEMA3C represses breast cancer cell migration and proliferation [19]. Another study found that SEMA3C induces symmetric divisions of breast cancer stem cells (CSC), which leads to the formation of spheres and expands the CSC population [20]. In addition, VEGF-C/NRP2 signaling enables papillary thyroid carcinoma cells to invade through the p38 mitogen-activated protein kinase (MAPK) signaling cascade [21]. Moreover, p38 isoforms are associated with breast cancer aggressiveness. Targeting p38δ suppresses cell proliferation in breast cancer cells and inhibiting p38α decreases breast cancer metastases [22,23]. However, the mechanism underlying NRP2, which plays an essential role in metastasis, remains elusive in CTCs.

This study sought to understand miRNA-dependent molecular phenotypes in CTC-mimicking suspension cells. We conducted small-RNA sequencing and performed integrative analyses of mRNA-miRNA sequencing. From these analyses, we identified a novel miR-146a-NRP2-axis that determines the invasive capacity of CTC-mimicking suspension cells. Moreover, we demonstrated that miR-146a plays an important role in reducing p38 signaling by regulating NRP2 expression. Finally, we showed that NRP2 expression levels are associated with the survival rates of breast cancer patients.

## 2. Materials and Methods

### 2.1. Cell Culture

MDA-MB-468 human breast cancer cell lines were grown in PRMI-1640 medium (HyClone, Marlborough, MA, USA) with 10% fetal bovine serum (FBS; HyClone, Marlborough, MA, USA) at 37 °C in a 5% CO_2_ incubator. In our previous study, MDA-MB-468 suspension cells were designed for a CTC-mimicking model [6]; there, 0.1 µg/mL nocodazole was applied to the MDA-MB-468 adherent cells for 16 h to synchronize cell mitosis. After being harvested and washed with phosphate-buffered saline (PBS), the cells were maintained on ultralow attachment plates (Corning, NY, USA). More than 160 cell passages were employed. HeLa cells were cultured in DMEM/HIGH GLUCOSE (DMEM; HyClone, Marlborough, USA); they were supplemented with 10% FBS, 1% penicillin-streptomycin (WelGENE, Daegu, Korea) and Mycozap (Lonza, Basel, Switzerland) in a 5% CO_2_ incubator at 37 °C.

### 2.2. Analysis of Small-RNA and mRNA Sequencing

Total RNAs including miRNAs, were extracted from MDA-MB-468 adherent and suspension cells, and small-RNA sequencing of the prepared library was conducted by Theragen Etex (Seoul, Korea). The quality of the sequencing data was analyzed by the Theragen Etex Bioinformatics Team. Differential expression and miRNA–mRNA interaction analyses were also performed. The RNA-seq data (PMID: 29416640) were reanalyzed, and differentially expressed genes (DEGs) were defined as those over a log2-fold change between adherent and suspension cells. The DEGs were applied to biological process prediction using Metascape (PMCID: PMC6447622).

### 2.3. Transfection

Lipofectamine RNAimax and Lipofectamine 2000 (Invitrogen, Carlsbad, CA, USA) were used for transfection, following the manufacturer’s instructions. AccuTarget™ *NRP2* siRNA, negative control siRNA, hsa-miR-146a mimic, and miRNA negative control mimic #1 were purchased from Bioneer (Daejeon, Korea) and transfected with Lipofectamine RNAimax. Transfection for both the *NRP2* 3′UTR cloning vector (Promega, Madison, WI, USA) and miRNA mimics was applied using Lipofectamine 2000. SEMA3C (R&D Systems, Minneapolis, MN, USA) was treated at 50 ng/mL at designated timepoints after 24 h starvation.

### 2.4. RNA Extraction and Quantitative Real-Time Polymerase Chain Reaction (PCR)

The total RNA was isolated using the miRNeasy RNA Isolation Kit (Qiagen, Hilden, Germany). Purified RNA was reverse transcribed using the Taqman miRNA Reverse Transcription Kit (Applied Biosystems, Foster City, CA, USA) for miRNA and the qPCRBIO cDNA Synthesis Kit (PCR Biosystems, London, UK) for mRNA. The miRNA quantitative real-time PCR (qRT-PCR) was performed using Taqman Universal Master Mix II, no UNG (Applied Biosystems, Foster City, CA, USA), and miR-146a was detected with Taqman probes. qRT-PCR was performed using qPCRBIO syGreen Mix Lo-ROX (PCR Biosystems, London, UK) according to the manufacturer’s instructions. RNU48 and ribosomal 18s were used as internal controls for quantifying miR-146a and *NRP2* mRNA, respectively. The primers used for qRT-PCR amplification were as follows: *NRP2* forward for, 5′-CTGTGGGTCATCCGTGAGGAC-3′, *NRP2* reverse for, 5′-ATGGGTTCCATGCAGTTCTCCAG-3′, *18S* forward for, 5′-ACCCGTTGAACCCCATTCGTGA-3′, and *18S* reverse for, 5′-GCCTCACTAAACCATCCAATCGG-3′.

### 2.5. Western Blotting

Cells were lysed with an RIPA buffer (Biosesang, Seongnam, Korea) containing a protease inhibitor cocktail (GenDEPOT, Barker, TX, USA), for which Phosphatase Inhibitor Cocktail 2 and 3 (Sigma Aldrich, Saint Louis, MO, USA) were used. Subsequently, cell lysates were centrifuged at 13,000 rpm and 4 °C for 15 min. Protein quantification was conducted using the Pierce BCA Protein Assay Kit (Thermo Fisher Scientific, Waltham, MA, USA), and the same protein concentration was boiled. Equal quantities of total proteins were loaded into sodium dodecyl sulfate-polyacrylamide gel and transferred to a polyvinyl difluoride membrane (Merck Millipore, Billerica, MA, USA). After blocking at 3% bovine serum albumin (BSA) in 0.2% PBS with Tween detergent (PBST), the protein bands were hybridized with primary antibodies overnight at 4 °C. Anti-GAPDH (#2118, Cell Signaling Technology, Danvers, MA, USA), anti-NRP2 (#AF2215, R&D Systems, Minneapolis, MA, USA), anti-p38 (#9212, Cell Signaling Technology, Danvers, MA, USA), and anti-phospho-p38 (Thr180/Tyr18) (#9211, Cell Signaling Technology, Danvers, MA, USA) were used to detect each protein. For immunodetection and development, the horseradish peroxidase (HRP)-conjugated secondary antibodies, anti-rabbit (#7074, Cell Signaling Technology, Danvers, MA, USA) and anti-goat (#ab6741, Abcam, Cambridge, MA, USA), were treated for 1 h at room temperature, and an enhanced chemiluminescent detection system (Thermo Fisher Scientific, Waltham, MA, USA) was employed.

### 2.6. Trans-Well Invasion Assay

Twenty-four trans-well plates (8.0 μm pore size; SPL, Pocheon, Korea) were coated with Matrigel (Corning, NY, USA) and diluted in serum-free media. For the invasion assay, transfected cells were seeded with serum-free media into the upper chamber, and complete growth media were placed in the bottom chamber. After 48–72 h at 37 °C in a 5% CO_2_ incubator, the media in the upper chamber were removed and washed with PBS. The cells were fixed in 4% formaldehyde and permeabilized with 100% methanol. The cells stained with 0.5% crystal violet (diluted in 20% methanol) were counted and analyzed using ImageJ software. To identify the effects of SEMA3C on the invasion assay, SEMA3C (50 ng/mL) was added to the upper chamber for 24 h before the media were removed therefrom.

### 2.7. Cell Migration Assay

After transfection, MDA-MD-468 adherent cell migration assay was performed using 12-well plates with SPLScar Block (SPL, Pocheon, Korea), according to the manufacturer’s instructions. Cells were maintained in a medium containing 10% FBS for 24 h; then they were observed under an optical microscope, and the differences were measured using ImageJ software.

### 2.8. Cell Proliferation Assay

To assess the proliferation ability, a cell proliferation assay was performed using cell-counting and WST-1 assays. After transfection, the cells were seeded in six-well plates to perform the cell-counting assay. After 2–3 day, cells were washed with PBS. 0.25%. Trypsin EDTA (WelGENE, Korea) was added for 2 min at 37 °C and then neutralized with complete growth media. The cells were diluted in trypan blue and counted daily. To evaluate the effect of SEMA3C on the proliferation assay, cells were seeded into serum-free media in six-well plates. After 24 h, the SEMA3C (50 ng/mL) was treated for 24 h, and the cells were counted with trypan blue staining. Treatment with 10% FBS was used as a positive control.

For the WST-1 assay, transfected cells were seeded at a density of 1 × 10⁴ cells/well into 96-well plates for 24–72 h. Ten microliters of EZ-Cytox (Deaillab, Seoul, Korea), a WST-1 reagent, was added into each well per day. After incubation for 30 min at 37 °C, the absorbance was measured at 450 nm.

### 2.9. Luciferase Reporter Assay

The human *NRP2* 3′UTR (3075 bp) that included the predicted miR-146a-binding seed sequences was cloned between the NOTI and XHOI sites of a psiCHECK-2 vector (Promega, Madison, WI, USA). The first AGTTCTC sequence in the seed sequences of *NRP2* was mutated to AACTCTA, and the second was mutated to AATCACC using the QuikChange II site-directed mutagenesis kit (Agilent, Santa Clara, CA, USA). HeLa cells were transfected with a luciferase reporter construct (containing either the wild or mutant type of *NRP2* 3′UTR) and miRNA (either miR-146a-mimicking or negative control miRNA) using Lipofectamine 2000. Forty-eight hours after transfection, the cells were lysed, and the luciferase activity was measured using the Dual-Luciferase Reporter Assay Kit (Promega, Madison, WI, USA) following the manufacturer’s instructions.

### 2.10. Insilico Analysis

The dataset of a Kaplan–Meier plotter was analyzed to determine the overall survival and distant metastasis-free survival of breast cancer patients with respect to the NRP2 expression level.

### 2.11. Statistical Analysis

All experiments were performed at least thrice and analyzed using GraphPad Prism 5.0 software. Unpaired two-tailed Student’s *t*-tests were conducted to assess statistical differences; a score of *p* < 0.05 was considered statistically significant. Values of * *p* < 0.05, ** *p* < 0.01, and *** *p* < 0.001 were regarded as norms of significant differences.

## 3. Results

### 3.1. Integrative Analysis of mRNA and Small–RNA Sequencings

**Figure 1:**
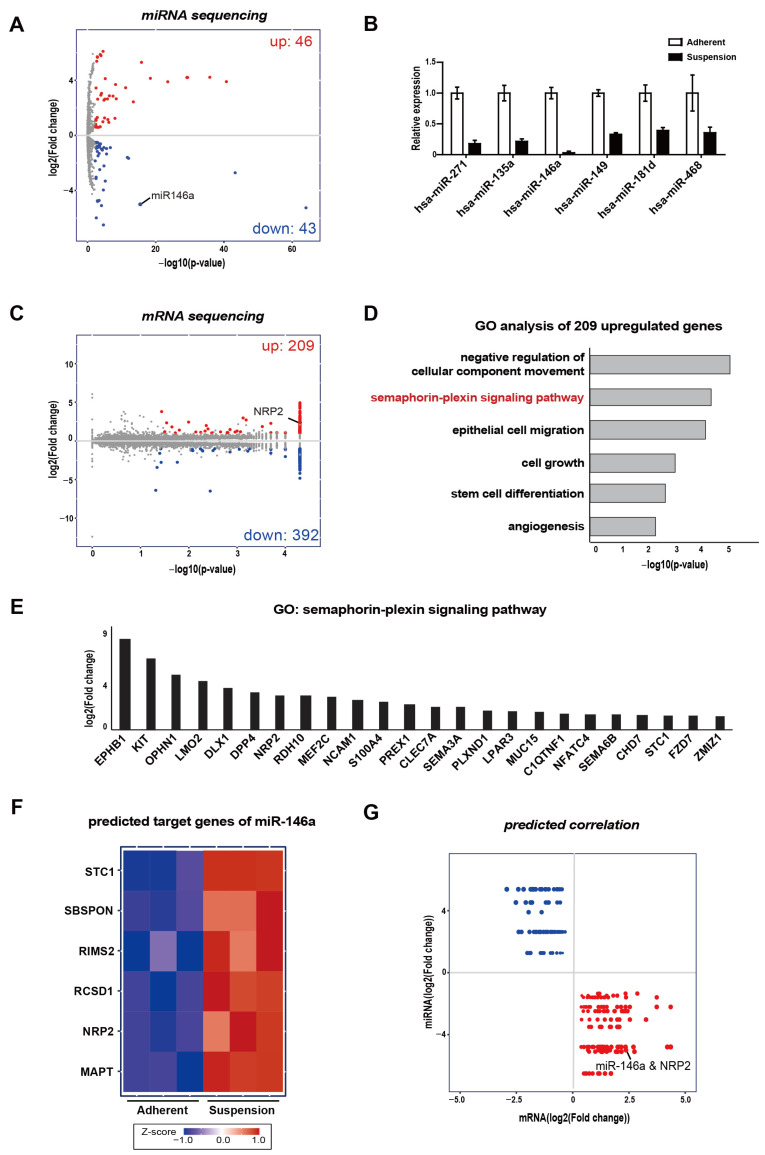
Integrative analyses of mRNA-miRNA sequencing showing differentially expressed miRNAs and the predicted targets of miR-146a. (**A**) Scatter plot showing 89 differentially expressed miRNAs (obtained through small-RNA sequencing) in MDA-MB-468 suspension cells compared to adherent cells. The threshold for differential miRNA expression (|FC| ≥ 1.2) and adjusted *p*-value (<0.05) is indicated on log2 and log10 scales. (**B**) Relative expressions of significantly downregulated miRNAs in adherent and suspension cells. (**C**) Scatter plot indicating 601 DEGs (obtained through mRNA sequencing) in suspension cells compared to adherent cells. The threshold for differential miRNA expression (|FC| ≥ 2) and adjusted *p*-value (<0.05) is indicated on log2 and log10 scales. (**D**) RNA-seq-based gene ontology analysis of 209 upregulated genes. (**E**) GO analysis of upregulated genes involved in semaphorin–plexin signaling pathway. (**F**) Heatmap of predicted miR-146a targets between adherent cells and suspension cells. (**G**) Scatter plot of fold changes for predicted correlation between mRNA and miRNA (Blue: high miRNA and low mRNA, Red: low miRNA and high mRNA).

To identify the miRNAs associated with the altered molecular phenotype of CTC-mimicking suspension cells, we performed small-RNA sequencing for MDA-MB-468 adherent cells and suspension cells. The results show that amongst the suspension cells, 46 miRNAs were upregulated and 43 were downregulated (Figure 1A). In these analyses, five upregulated and six downregulated miRNAs had significantly differential expressions in the suspension cells (Table 1). In particular, miR-146a showed the largest reduction in suspension cells compared to adherent cells (Figure 1B). Furthermore, miR-146a has been reported to act as a tumor suppressor in many types of cancer [24,25,26]. Therefore, we focused on miR-146a, which shows the most altered miRNA expression.

Next, we analyzed the RNA sequencing to identify the predicted targets of miRNAs. We found that 209 increased, and 392 decreased genes were differentially expressed in the suspension cells (Figure 1C). First of all, we focused on the increased genes to find out the relevance to tumor aggressiveness. Through gene ontology analysis of 209 upregulated genes, we found that the semaphorin–plexin signaling pathway was considerably enhanced in the CTC-mimicking suspension cells (Figure 1D); 24 upregulated genes were related to the semaphorin–plexin signaling pathway (Figure 1E). Among the significantly upregulated 209 mRNAs in suspension cells, six genes were predicted as possible targets of miR-146a using TargetScan (Figure 1F and Table 2).

Of these six genes, NRP2 and STC1 are involved in the semaphorin–plexin signaling pathway. NRP2 showed more prominent differential expression levels and is known to interact with the semaphorin family as an oncogenic gene in many tumors [27,28,29]. MiR-146a and NRP2 were inversely correlated in suspension cells, indicating that suspension cells feature downregulated miR-146a and upregulated NRP2 expression levels (Figure 1G). Consequently, RNA–miRNA sequencing identified the potential miRNAs involved in the molecular phenotype of CTC-mimicking suspension cells.

### 3.2. MDA-MB-468 Suspension Cells Show Upregulated NRP2 and Downregulated miR-146a Expressions Compared to Adherent Cells

**Figure 2:**
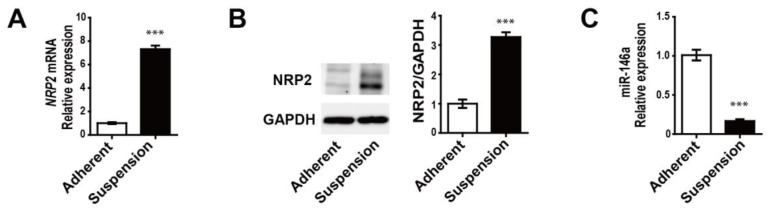
Increased NRP2 expression levels and decreased miR-146a expression levels in MDA-MB-468 suspension cells, according to the mRNA and miRNA sequencing analysis. (**A**) *NRP2* mRNA expression in adherent cells and suspension cells. (**B**) NRP2 protein expression in adherent cells and suspension cells. (**C**) MiR-146a expression in adherent cells and suspension cells. *** *p* < 0.0001 compared to controls calculated via an unpaired two-tailed Student’s *t*-test. Error bars denote the standard error of the mean.

To validate that the NRP2 expression levels were inversely correlated with miR-146a expression, we performed real-time quantitative PCR and Western blotting for MDA-MB-468 adherent and suspension cells. Both the mRNA and protein expressions of NRP2 increased in suspension cells compared to those in adherent cells (Figure 2A,B, respectively), whereas miR-146a expression levels were dramatically decreased in suspension cells (Figure 2C). These results suggest a strong negative correlation between NRP2 and miR-146a in adherent and suspension cells.

### 3.3. MiR-146a Directly Targets NRP2 and Regulates SEMA3C-Induced Phosphorylation of p38

**Figure 3:**
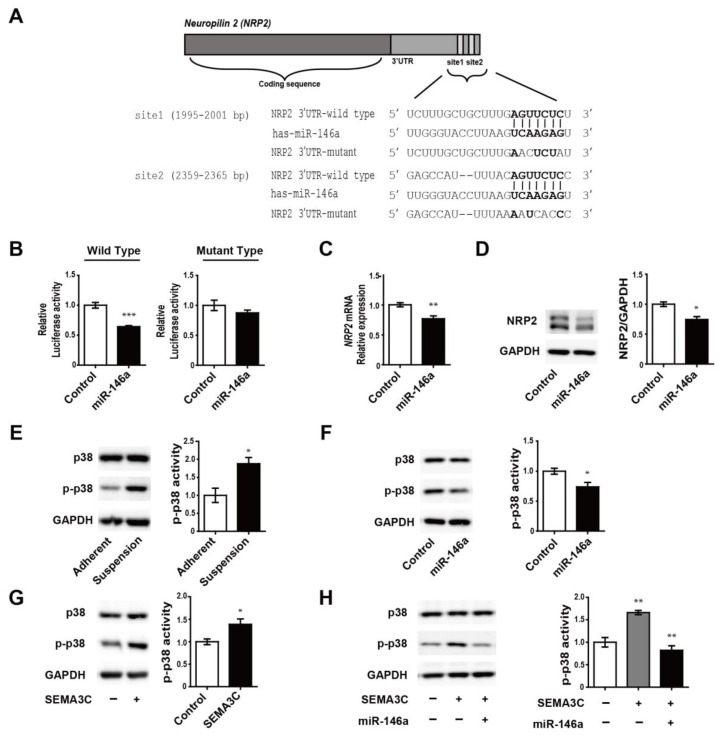
MiR-146a regulation of NRP2 expression (through binding *NRP2* 3′UTR) and reduction in SEMA3C-mediated phosphorylation of p38 (p-p38) level. (**A**) Predicted sequences of *NRP2* 3′UTR targeted by miR-146a and mutated sequences to disrupt the recognition of miR-146a. (**B**) Luciferase activity after the overexpression of miR-146a in HeLa cells transfected with a reporter construct containing either the wild-type or mutagenized type 3′UTR of *NRP2*. (**C**,**D**) NRP2 mRNA and protein expression in response to the overexpression of miR-146a in MDA-MB-468 suspension cells. (**E**) Activity of p-p38 in adherent and suspension cells (**F**) Activity of p-p38 in response to the overexpression of miR-146a in suspension cells. (**G**) Activity of p-p38 after treatment of suspension cells with SEMA3C (50 ng/mL). (**H**) Activity of p-p38 in response to SEMA3C (50 ng/mL) overexpression miR-146a or non-overexpression control culture in suspension cells. * *p* < 0.01, ** *p* < 0.001, and *** *p* < 0.0001 compared to controls calculated via an unpaired two-tailed Student’s *t*-test. Error bars denote the standard error of the mean.

We further investigated the relationship between miR-146a and NRP2. To determine whether miR-146a directly regulates NRP2 expression, we first examined the potential that the 3′UTR of *NRP2* mRNA is targeted by the miR-146a, using a combination of algorithm software (e.g., TargetScan and miRbase.org). We found that miR-146a, which has highly conserved seed sequences, is predicted to bind to the 3′UTR of *NRP2* at two binding sites, suggesting that miR-146a may be a key regulator of NRP2 expression (Figure 3A). Next, we showed that cotransfection of the wild-type *NRP2* 3′UTR reporter construct with miR-146a mimics significantly reduced the luciferase activity; meanwhile, luciferase activity in cells transfected with mutant *NRP2* 3′UTR, which mutagenized both predicted binding sites of miR-146a, was unaltered (Figure 3B). Overexpression of miR-146a in MDA-MB-468 suspension cells led to a decrease in both the mRNA and protein expression of NRP2 (Figure 3C,D, respectively). These results indicate that miR-146a directly regulates NPR2 expression in suspension cells. We further found phosphorylation of the p38 (p-p38) MAPK, which is related to breast cancer metastasis [30,31] was increased in suspension cells compared to adherent ones (Figure 3E). Moreover, the overexpression of miR-146a in suspension cells was found to downregulate p-p38 levels (Figure 3F). The activity of p-p38 was increased after treatment with SEMA3C, which activated downstream signaling as a ligand of NRP2 (Figure 3G); furthermore, SEMA3C-induced p-p38 was decreased in response to the overexpression of miR-146a (Figure 3H). From these findings, we conclude that miR-146a may play an important role in the regulation of the p38 signaling pathway by targeting NRP2.

### 3.4. NRP2 Inhibition Attenuates Proliferation, Migration, and Invasion of MDA-MB-468 Cells

**Figure 4:**
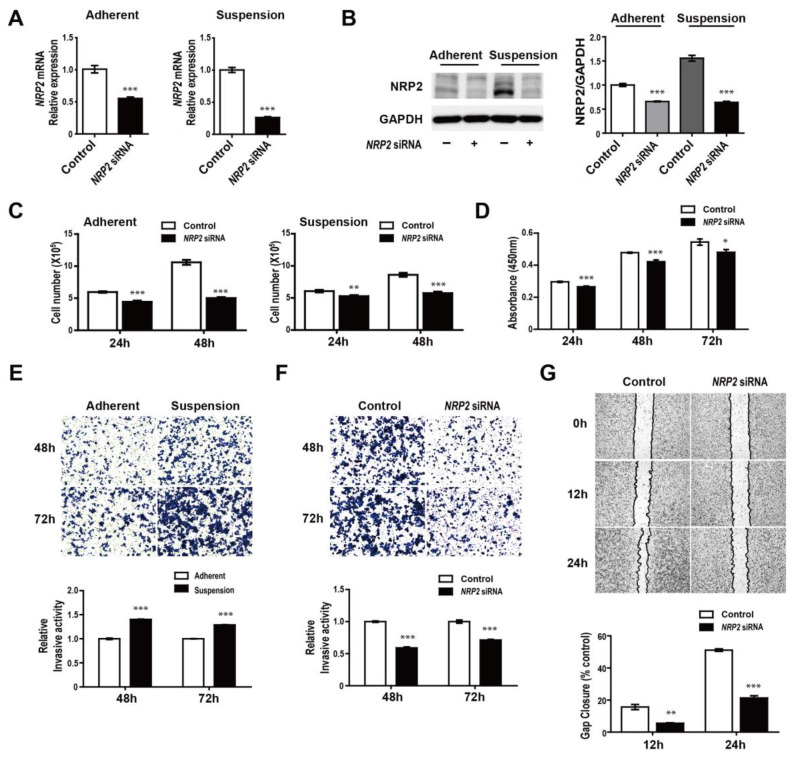
Effects of NRP2 knockdown on proliferation, migration, and invasion of MDA-MB-468 cells. (**A**,**B**) The NRP2 mRNA and protein expressions in adherent and suspension cells in response to NRP2 knockdown. (**C**) Cell proliferation assays in adherent and suspension cells transfected with *NRP2* siRNA or negative control RNA. (**D**) WST-1 assays in adherent cells after NRP2 knockdown. (**E**) Trans-well invasion assays in adherent and suspension cells. (**F**) Trans-well invasion assays in suspension cells transfected with *NRP2* siRNA or negative control RNA. (**G**) Migration assays in adherent cells transfected with *NRP2* siRNA or negative control RNA. * *p* < 0.01, ** *p* < 0.001, and *** *p* < 0.0001 compared to controls calculated via an unpaired two-tailed Student’s *t*-test. Error bars denote the standard error of the mean.

Previous studies have reported that NRP2 is associated with cancer metastasis [32], and we also found that NRP2 was considerably increased in suspension cells. To characterize the effects of NRP2 on tumorigenesis, we first determined the effects of the knockdown of NRP2. NRP2 mRNA and protein levels were markedly reduced when transfected with *NRP2* siRNA in MDA-MB-468 adherent and suspension cells (Figure 4A,B, respectively). Next, we investigated whether the knockdown of NRP2 affected functional phenotypes in the adherent and suspension cells. We found that both adherent and suspension cells exhibited a decreased proliferative ability in response to NRP2 knockdown, indicating that the divided cells’ numbers were lower in cells with *NRP2* siRNA than in the negative control siRNA (Figure 4C,D). Next, we tested the invasive capacities of the suspension cells. The suspension cells showed enhanced invasion abilities compared to the adherent ones (Figure 4E); furthermore, the knockdown of NRP2 considerably repressed the invasion (Figure 4F). As shown in Figure 4G, migration was suppressed when NRP2 knockdown took place in adherent cells. Overall, these findings suggest that regulation of NRP2 expression may be a key therapeutic option in breast cancer.

### 3.5. MiR-146a Impairs SEMA3C-induced Prometastatic Phenotypes in MDA-MB-468 Suspension Cells

**Figure 5:**
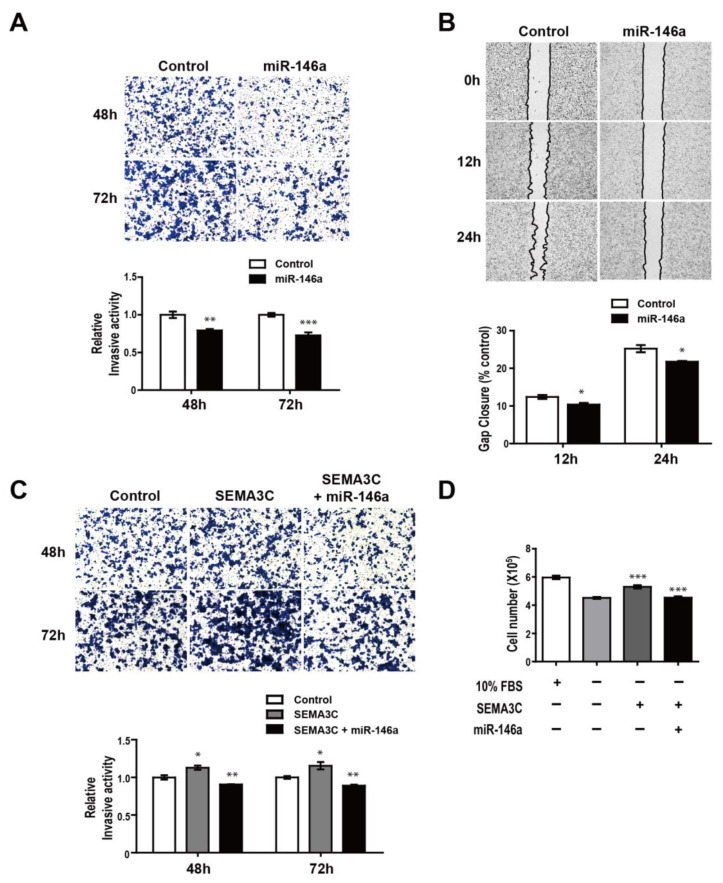
MiR-146a suppression of SEMA3C-induced proliferation and invasion in MDA-MB-468 suspension cells. (**A**) Trans-well invasion assay response to the overexpression of miR-146a in suspension cells. (**B**) Migration assays in adherent cells after the restoration of miR-146a. (**C**) Trans-well invasion assays in response to SEMA3C (50 ng/mL) overexpressed miR-146a or non-overexpression control cultures in suspension cells. (**D**) Cell proliferation assays after treatment with SEMA3C (50 ng/mL) overexpressed miR-146a or non-overexpression control cultures in suspension cells. * *p* < 0.01, ** *p* < 0.001, and *** *p* < 0.0001 compared to controls calculated via an unpaired two-tailed Student’s *t*-test. Error bars denote the standard error of the mean.

Given that the oncogenic functions of NRP2 activate many signaling pathways by binding with SEMA3C, we investigated the role of miR-146a (a regulator of NRP2 expression) in breast cancer aggressiveness. MiR-146a alleviated invasion in MDA-MB-468 suspension cells, reducing the number of invasive cells in response to the overexpression of miR-146a (Figure 5A). Moreover, the migratory abilities of adherent cells were decreased after the restoration of miR-146a expression (Figure 5B). We further examined the effects of miR-146a on SEMA3C-induced invasion. SEMA3C induced invasion in suspension cells and was completely abrogated when miR-146 was overexpressed (Figure 5C). MiR-146a also inhibits SEMA3C-mediated proliferation (Figure 5D). Treatment with 10% FBS was used as a positive control. These findings demonstrate that miR-146a inhibits SEMA3C-induced invasion and proliferation in suspension cells, suggesting the possibility that miR-146a may be an important factor in attenuating breast cancer CTCs.

### 3.6. Breast Cancer Patients with Increased NRP2 Are Associated with Poor Clinical Outcome

**Figure 6:**
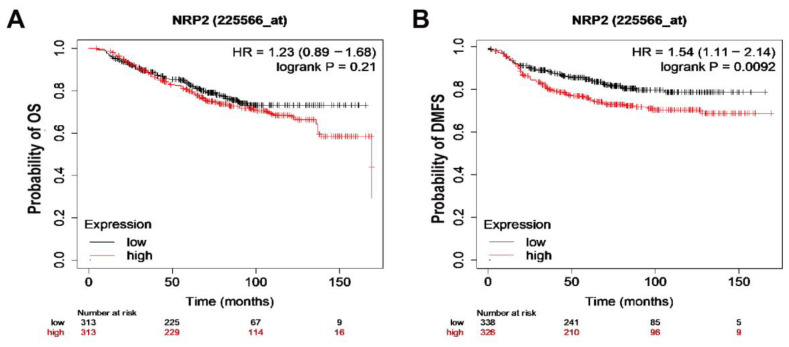
NRP2 expressions with respect to survival rates of breast cancer patients. (**A**) OS (*n* = 626, number of events = 313, *p* = 0.21) and (**B**) DMFS (*n* = 664, number of events = 326, *p* = 0.0092) for breast cancer patients with high NRP2 expression in the dataset, depicted on Kaplan–Meier plotter.

To further clarify the role of NRP2 in breast cancer survival, we evaluated the breast cancer case data from The Cancer Genome Atlas (TCGA). While no significant differences were observed in overall survival (OS) with respect to NRP2 expression (*p* = 0.21; Figure 6A), patients with high NRP2 expression exhibited significantly worse distant metastasis-free survival (DMFS) rates than those with low NRP2 expression (*p* = 0.0092; Figure 6B), suggesting the role of NRP2 as a novel biomarker for predicting recurrence in breast cancer patients. Although we could not analyze the association between low miR-146a expression and DMFS due to the unavailability of public data, these analyses further support the hypothesis that the miR-146a-NRP2 axis can be used in potential therapeutic strategies.

## 4. Discussion

CTCs are regarded as key metastatic factors; they detach from the primary tumor and enter the bloodstream. Detection of CTC clusters in circulation is associated with poor clinical outcomes for various cancers [7]. The CTC properties that contribute to the occurrence of metastatic lesions are considered important; however, their molecular phenotype remains incomplete. CTCs are estimated to comprise only 1–10 cells per 10 mL of blood in patients with tumors; thus, their isolation is challenging [33,34]. In particular, the alteration of miRNA expression in CTCs is not fully understood, though miRNA is a major regulator of tumorigenic processes. This study demonstrates that miR-146a plays a crucial role in suppressing the SEMA3C-induced invasive abilities of CTCs, by regulating NRP2. Four central findings can be reported: (i) five upregulated and six downregulated miRNAs were revealed by integrative analyses of miRNA–mRNA sequencing; (ii) the MiR-146a directly targets NRP2, which is associated with tumor aggressiveness; (iii) MiR-146a inhibits SEMA3C-induced phosphorylation of p38 (p-p38) and invasion in suspension cells; (iv) high NPR2 is associated with worse prognosis in breast cancer patients.

Previous studies have developed numerous ways to isolate CTCs, such as CTC-iChip, array-comparative genomic hybridization (CGH), and CRISPR activation screens (CRISPRas). Single CTCs have been separated from whole-blood samples using CTC-iChip by depleting white blood cells and identifying EpCAM- and HER2-stained cells [35]. Another study conducted array-CGH and next-generation sequencing, and they managed to detect CTCs in 21 out of 37 colorectal carcinoma patients [36]. Another study proposed CRISPRa, which uses gene-specific transcriptional activation to identify up-regulated genes in CTCs [37]. These studies identified several genes from the results of their innovative methods; however, they focused on determining the DEGs in CTCs. Recently, as the significance of regulatory miRNAs in cancer metastasis has become apparent, attempts have been made to profile mRNA and miRNA expressions. One study identified increased numbers of mRNAs and miRNAs (55 and 10, respectively) in CTCs obtained from breast cancer patients [38]. Moreover, miR-106b levels were considerably higher in the CTCs of breast cancer patients with poor OS rates [39]. Nevertheless, further studies of miRNAs are needed to suppress metastatic CTCs.

In this study, we employed MDA-MB-468 suspension cells as an alternative. By using CTC-mimicking suspension cells, we consistently maintained the suspension cells and performed numerous functional studies. Small-RNA sequencing was conducted to identify miRNAs involved in diminishing suspension cells. Six decreased miRNAs were observed, including miR-146a. Notably, miR-146a was significantly downregulated, indicating it may be a major regulator of metastatic suspension cells. In addition, a potential relationship between miR-146 and NRP2 was found.

It has been reported that NRP2 and miR-146a are related to cancer metastasis. Similarly, circulating CSCs (considered a small subset of CTCs) that are capable of self-renewal have attracted research attention [40]. NRP2, a coreceptor of autocrine VEGF signaling, plays an essential role in the proliferation and survival of breast CSCs. VEGF-NRP2 signaling contributes to focal adhesion kinase (FAK)-mediated Rac1 activation and inhibits large tumor suppressor kinases (LATS), leading to increased mammosphere formation of breast CSCs [41]. In a different study, NRP2 signaling exhibited enhanced tumor initiation via stimulation of the α6β1 integrin and FAK-mediated Ras/MEK signaling. Increasing NRP2 promoted the expression of GLI1, a hedgehog effector, which then induced BMI-1, a crucial stem cell factor [42]. Furthermore, several studies have reported that miR-146a suppresses tumor progression. It is known that miR-146a/b acts as a negative regulator of constitutive NF-κB activity. MDA-MB-231 breast cells showed markedly impaired invasion and migration ability expressing via the reduction in constitutive NF-κB activity by a high level of miR-146a expression [43]. In addition, the overexpression of miR-146a reduced the capacity of breast cancer cells to migrate and invade owing to the downregulation of RhoA [44].

## 5. Conclusions

In summary, many studies have indicated that NRP2 and miR-146a may prevent the deterioration of breast cancer. However, the role of the miR-146a-NRP2 axis in CTCs has remained elusive. We determined that the overexpression of miR-146a decreases NRP2 expression and p-p38 activity in suspension cells. Moreover, miR-146a attenuated the invasion and proliferation induced by SEMA3C (a ligand of NRP2), suggesting that miR-146a regulates the NRP2 signaling associated with oncogenic effects. Future investigations using miR-146a inhibitors and in vivo experiments with stably expressing miR-146a suspension cells should provide deeper insight into suspension cell invasion, because knockdown of pro-metastatic gene SCT1 reduces invasive capacity in MCF-7 cells and MAPT increases the metastases of breast CTCs [45,46].

We demonstrated that miR-146a is an important regulator in invasive breast cancer, regulating NRP2 expression. This study determined a novel correlation between miR-146a and p-p38 in CTC-mimicking suspension cells. Our current study proposes innovative therapeutic strategies for CTCs by suggesting miR-146a as a key diagnostic biomarker for breast cancer patients.

## Figures and Tables

**Table 1:** Summary of significantly differentially expressed miRNAs in suspension cells compared to adherent cells.

miRNA	Log 2-Fold Change	*p*-Value
**Upregulated miRNA**		
miR-187	3.90	$1.92\times 10^{-41}$
miR-224	1.27	$9.69\times 10^{-6}$
miR-488	5.38	$1.91\times 10^{-3}$
miR-582	2.63	$1.01\times 10^{-3}$
miR-1298	4.53	$4.43\times 10^{-2}$
**Downregulated miRNA**		
miR-135a	−2.21	$1.46\times 10^{-5}$
miR-146a	−5.00	$3.33\times 10^{-16}$
miR-149	−1.60	$2.06\times 10^{-12}$
miR-181d	−1.35	$2.58\times 10^{-6}$
miR-486	−1.49	$8.06\times 10^{-4}$
miR-1271	−2.48	$2.57\times 10^{-4}$

**Table 2:** Predicted targets of upregulated miR-146a in MDA-MB-468 suspension cells.

Symbol	Gene Name	Log 2-Fold Change
STC1	Stanniocalcin 1	1.20
SBSPON	Somatomedin B and thrombospondin type 1 domain containing	2.20
RIMS2	Regulating synaptic membrane exocytosis 2	1.54
RCSD1	RCSD domain containing 1	1.98
NRP2	Neuropilin 2	3.02
MAPT	Microtubule associated protein tau	3.30
